# Pulmonary hemorrhage in a patient with Marfan syndrome

**DOI:** 10.36416/1806-3756/e20240217

**Published:** 2024-11-16

**Authors:** Matheus de Almeida Costa, Miriam Menna Barreto, Edson Marchiori

**Affiliations:** 1. Universidade Federal do Rio de Janeiro, Rio de Janeiro (RJ) Brasil.

**Figure 1:**
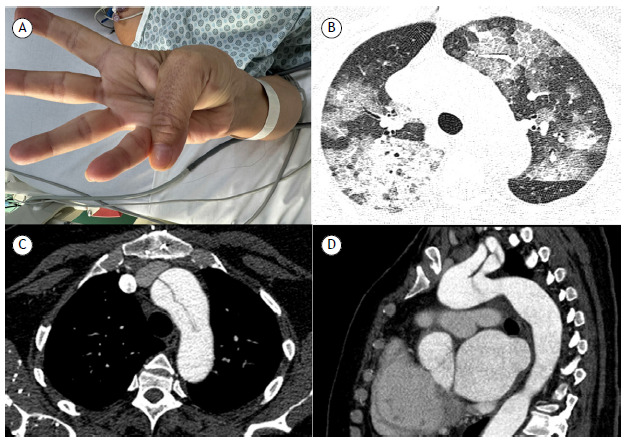
In A, clinical photograph showing hyperextensibility of the thumb. Note that the distal phalanx of the thumb extends beyond the ulnar border of the hand. In B, axial chest CT image (lung window) showing bilateral areas of ground-glass attenuation and interlobular septal thickening (a crazy-paving pattern). In C and D, respectively, axial and sagittal reconstructions (mediastinal window) showing areas of aortic dilatation and aortic stenosis, particularly in the ascending portion, as well as aortic dissection.

A 47-year-old woman was diagnosed with Marfan syndrome 10 years previously. At diagnosis, she was noted to have joint hyperextensibility (Figure 1A); aortic dilatation and dissection (Figures 1B and 1C); and aortic valve involvement. She underwent surgical correction and progressed well. She had been taking an oral anticoagulant (warfarin) since then. She presented to our service with a ~1-week history of cough with hemoptysis and hematuria. A chest CT scan showed bilateral ground-glass opacities (Figure 1D). Alveolar hemorrhage secondary to warfarin-induced coagulopathy was suspected and subsequently confirmed by bronchoscopy. The prothrombin time/international normalized ratio was 7.1. It was reversed with two units of fresh frozen plasma and intravenous vitamin K. Respiratory support was provided, and the patient was discharged from the hospital 20 days later in asymptomatic condition. 

Marfan syndrome is an inherited connective tissue disorder that may affect various systems, including the cardiovascular, musculoskeletal, ocular, and pulmonary systems. Common cardiovascular manifestations, most of which are substantial contributors to mortality, include the following: aortic root dilatation with or without aortic valve insufficiency; aortic dissection; aortic aneurysm; and pulmonary artery dilatation. Pulmonary manifestations include paraseptal emphysema with upper lobe bullae and spontaneous pneumothorax. Patients with Marfan syndrome and cardiovascular alterations are frequently treated with warfarin for anticoagulation, which makes them susceptible to pulmonary hemorrhage, as seen in our patient.^1^ ^-^ ^3^
